# Non-Invasive Monitoring of Ethanol and Methanol Levels in Grape-Derived Pisco Distillate by Vibrational Spectroscopy

**DOI:** 10.3390/s21186278

**Published:** 2021-09-18

**Authors:** Ahmed Menevseoglu, Didem P. Aykas, Beatriz Hatta-Sakoda, Victor Hugo Toledo-Herrera, Luis E. Rodriguez-Saona

**Affiliations:** 1 Department of Food Engineering, Faculty of Engineering and Natural Sciences, Gumushane University, Gumushane 29100, Turkey; amenevseoglu@gumushane.edu.tr; 2 Department of Food Engineering, Faculty of Engineering, Adnan Menderes University, Aydin 09100, Turkey; didem.cinkilic@adu.edu.tr; 3 Facultad de Industrias Alimentarias, Universidad Nacional Agraria La Molina, Av. La Molina s/n, La Molina, Lima 15024, Peru; bhs@lamolina.edu.pe; 4 Destileria 16, Fundo San Nicolas Lote P5 Villacuri y Lanchas-Pisco, Ica 11600, Peru; victorhugoth@gmail.com; 5 Department of Food Science and Technology, The Ohio State University, 110 Parker Food Science and Technology Building, 2015 Fyffe Road, Columbus, OH 43210, USA

**Keywords:** Pisco, ethanol, methanol, adulteration, Raman, FT-IR

## Abstract

Handheld Raman and portable FT-IR spectroscopy devices were evaluated for fast and non-invasive determination of methanol and ethanol levels in Peruvian Pisco. Commercial Peruvian Pisco (*n* = 171) samples were kindly provided by the UNALM Alliance for Research in Alcohol and its Derivatives (Lima, Peru) and supplemented by purchases at grocery and online stores. Pisco spectra were collected on handheld Raman spectrometers equipped with either a 1064 nm or a 785 nm excitation laser and a portable infrared unit operating in transmission mode. The alcohol levels were determined by GC–MS. Calibration models used partial least-squares regression (PLSR) to develop prediction algorithms. GC–MS data revealed that 10% of Pisco samples had ethanol levels lower than 38%, indicating possible water dilution. Methanol levels ranged from 10 to 130 mg/100 mL, well below the maximum levels allowed for fruit brandies. Handheld Raman equipped with a 1064 nm excitation laser gave the best results for determining ethanol (SEP = 1.2%; RPre = 0.95) and methanol (SEP = 1.8 mg/100 mL; RPre = 0.93). Randomly selected Pisco samples were spiked with methanol (75 to 2800 mg/100 mL), and their Raman spectra were collected through their genuine commercial bottles. The prediction models gave an excellent performance (SEP = 98 mg/100 mL; RPre = 0.97), allowing for the non-destructive and non-contact determination of methanol and ethanol concentrations without opening the bottles.

## 1. Introduction

Counterfeiting of spirit drinks is a worldwide problem done in many forms such as substitution, stretching with lower-grade products, or creating counterfeits with industrial, surrogate, or locally produced alcohols [1]. Wine (Brandy) and pomace distilled spirit products are well appreciated and valorized liquors worldwide [2]. Brandy is a spirit drink produced from wine spirit, whether or not blended with distillate and matured for at least one year in oak receptacles, producing changes in the composition of the spirit important for its quality (taste, flavor, and color) of the final products [3]. Brandies are produced in almost every wine-producing region (i.e., Armagnac, Cognac, Jerez) and are differentiated by provenance, grape cultivar, and type of still used [4]. To reduce the production costs, water or ethanol is sometimes used for counterfeiting brandy, leading to unfair trading practices. Other distilled grape products, derived from pomace-based wines, include marc (France), grappa (Italy), aguardiente or orujo (Spain), Pisco (Peru), bagaceira (Portugal), and tsipouro (Greece) [5].

The most known South American brandy is Pisco, made mainly from Muscat grapes and having economic and cultural importance, with its production dating to the 16th century [6]. Grape varieties employed for Pisco are classified into non-aromatic (Quebranta, Mollar, Negra Criolla, Uvina) and aromatic (Italia, Torontel, Albilla, Moscatel), giving Pisco a unique flavor profile (i.e., alcohols, terpenes, aldehydes, ketones, esters, phenols, furfural) associated with its signature aroma and taste [7]. The increase in production of this grape distillate drink has unfortunately led to economic adulteration, mainly by water dilution, replacing ethanol with the cheaper methanol to increase profits, or adding glycerol to increase sweetness [8]. Methanol is present in brandies, regardless of the fruit raw material (i.e., grape, plum, apple), and determines its authenticity and level of safety for consumption. Methanol in alcoholic beverages is formed by the enzymatic hydrolysis of the methoxyl groups of pectins during fruit fermentation, and its levels depend on the concentration of pectin in the fruit, the degree of pectin methoxylation, the degree of rotting of the fruit, and pectinesterase activity [5].

Conventional analytical techniques such as gas chromatography (GC), high-performance liquid chromatography (HPLC), capillary electrophoresis, and Nuclear Magnetic Resonance (NMR) have focused on certain marker compounds in authentic brandy products. Although highly sensitive, these methods require transportation of the samples to analytical facilities, are destructive, time-consuming, expensive, and require complex sample preparation [1,3]. Vibrational spectroscopic methods allow for simple operation, low-cost, and sensitivity, providing convenient platforms for effective surveillance of alcoholic spirit beverages and curtailing the risk of illicit ingredients being added for economic gain or of contaminants formed during inadequate processing. Nagajaran et al. (2006) reported the determination of methanol in alcoholic beverages by an attenuated total reflectance (ATR) technique using a calibration model developed with a synthetic sample set and reporting a variation of ±0.4% in predicting a test set [9]. The development of handheld MIR, Raman, and NIR spectrometers has drastically impacted the ability of end-users to conduct field-based as well as in situ analyses, allowing the rapid and non-destructive identification of unknown samples. Thus, portable instruments provide the ability to make informed decisions on the spot [9]. Portable MIR spectrometers (based on Michelson interferometers) operating in the attenuated total reflection (ATR) or diffuse reflection modes are based on small versions of conventional interferometer designs that are temperature-, shock-, and vibration-insensitive, displaying similar performance to bench-top instruments in field applications [10,11]. On the other hand, miniaturized Raman systems are offered in a broad range of configurations, with different excitation sources (532, 785, and 1064 nm) and operational features. A single-excitation Raman system can be miniaturized in a single unit as small as a cellular phone [12]. Key to the miniaturization of Raman systems have been advances in compact and efficient volume phase grating (VPG) and high-power solid-state lasers generating narrow lines. This research takes advantage of the inherent selectivity of mid-infrared (MIR) and Raman spectroscopy that provides access to fingerprint spectra for probing complex samples in the presence of interfering components [11].

The aim of the study was to evaluate handheld/portable technologies for the rapid detection of the ethanol and methanol content of Pisco. This study also evaluated the performance of a handheld Raman spectrometer in the determination of methanol concentration through commercial Pisco bottles.

## 2. Materials and Methods

Pisco samples (*n* = 171) were obtained from local grocery stores in Peru, online sales, and provided by Ms. Hatta-Sakoda from the Universidad Nacional Agraria La Molina (Lima, Peru) and Mr. Victor Toledo (Pisco 1615 Inc., Ica, Peru). An effort was made to collect Pisco samples from different producers, from rustic operations to large distilleries. The samples represented a wide selection of Pisco spirits manufactured with different grapes (Quebranta (*n* = 67), Italia (*n* = 44), Torontel (*n* = 25)), from different locations (Cañete and Ica), and with grape blends (“acholados” (*n* = 25)) and green must or specialty Pisco distilled when there is still sugar present in the must (*n* = 10).

### 2.1. Determination of Ethanol and Methanol Concentrations by GC–MS

Alcohol (ethanol and methanol) levels in Pisco samples were determined using an Agilent Technologies 7820A GC equipped with a 5877B MSD (Santa Clara, CA, USA) single quadrupole mass spectrometry detector. A DB-WAX UI column (Agilent Technologies, Santa Clara, CA, USA), 30 m × 250 µm (internal diameter) × 0.25 µm (film thickness) was used for the analysis. The sample injection volume into the GC was 0.3 µL under split mode at a 20:1 ratio under a constant flow of 1 mL/min on a column with an injection port temperature of 220 °C. The conditions for separation included an initial oven temperature set at 50 °C for 3 min, and then the oven temperature was increased at a rate of 4 °C/min to 80 °C and held for 5 min, followed by a temperature increase at a rate of 15 °C/min, reaching a final temperature of 200 °C and held for 1 min. The mass spectrometer source temperature was set at 240 °C, and the quadrupole temperature was set at 180 °C. The electron impact ionization of the MS was set at 70 eV and was operated in SIM and SCAN mode with a mass range of 30 to 250 atomic mass units (amu). The reference ion (m/z) used to identify and quantify ethanol and methanol was set for 45 m/z and 31 m/z, respectively. A calibration curve using pure ethanol and methanol standards was developed using solutions with concentrations ranging from 25 to 45% *v/v* ethanol in water and from 0.03 to 1% *v*/*v* methanol in 40/60 ethanol/water solutions.

### 2.2. Vibrational Spectroscopy

**Figure 1:**
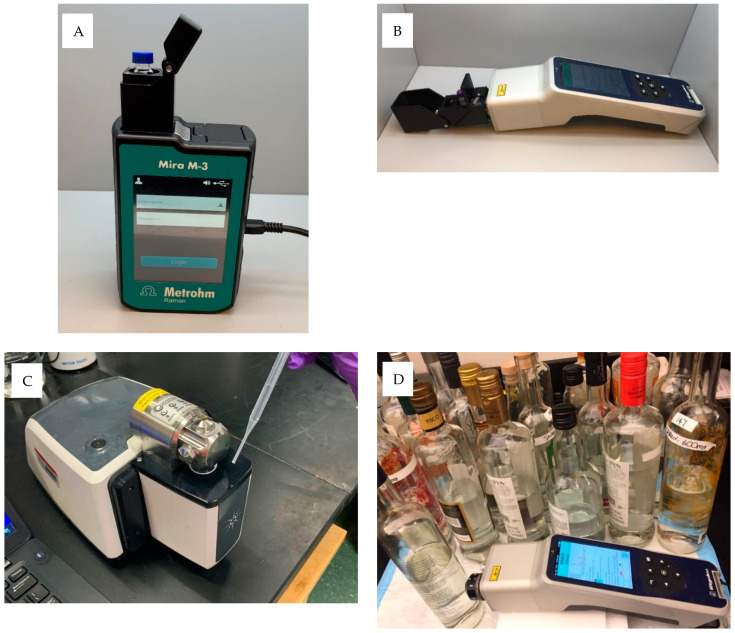
Vibrational spectroscopy units used in this research. (**A**) Metrohm Mira M3 (785 nm) Raman instrument, (**B**) handheld Progeny Rigaku (1064 nm), (**C**) portable Agilent Cary 630 FT-IR device, and (**D**) handheld Progeny Rigaku (1064 nm) instrument conducting the through-the-bottle analysis.

Raman spectra from Pisco samples were directly collected from the liquid by placing it in 2 mL glass vials. Raman spectra were collected using the handheld Mira M3 (Metrohm Inc., Herisau, Switzerland) (Figure 1A) and Progeny (Rigaku Corporation, Wilmington, MA, USA) (Figure 1B) systems. The Mira M3 spectrometer was equipped with 100 mW output power and a 785 nm excitation laser, and data were collected in the range from 400 cm^−1^ to 2300 cm^−1^. The Mira M3 is equipped with orbital raster scanning (ORS) that increases sample volume interrogation, delivering a high spectral resolution across a large sample area. The Progeny spectrometer operated a 1064 nm excitation laser with a 300 mW output power and a thermoelectric cooled Indium–Gallium Arsenide (InGaAs) array detector; the data were collected at 8 cm^−1^ resolution with 15 co-scans in the Raman shift range between 200 and 2500 cm^−1^ using the Progeny 2.0 software.

Mid-infrared spectra were collected on a portable Cary 630 Fourier Transform Mid-infrared (FT-IR) spectrometer (Agilent Technologies Inc., Santa Clara, CA, USA) equipped with a dial-path transmittance accessory set at 50 μm path length (Figure 1C). The equipment employed a Zinc Selenide (ZnSe) beam splitter and a deuterated triglycine sulfate (DTGS) detector. A total of 100 µL of Pisco sample was directly deposited onto the dial path accessory opening, and a background spectrum was collected before every spectral measurement to eliminate any possible environmental effect. Spectra were collected in the range between 4000 and 700 cm^−1^ with a resolution of 4 cm^−1^, and 64 scans were co-added to improve the signal-to-noise ratio. Duplicate independent spectral measurements were taken for each sample, and spectra were recorded using Agilent MicroLab PC software (Agilent Technologies Inc., Danbury, CT, USA).

### 2.3. Methanol Spiking and Spectroscopy Measurements

Pisco samples (*n* = 64) were randomly selected and spiked with methanol at concentrations ranging from 75 to 2800 mg/100 mL in absolute alcohol, resulting in a total of 116 spiked samples. The methanol range (75 to 2800 mg/100 mL) was selected to cover the legal limits and the reported methanol adulteration levels in alcoholic beverages leading to intoxication [13,14,15]. Spiked (*n* = 116) and pure Pisco samples (*n* = 64) were transferred into 2 mL glass vials, and Raman spectral measurements were carried out using the handheld Progeny Raman equipment. Besides using glass vials, spectra from randomly selected pure (*n* = 33) and spiked Pisco samples (*n* = 29) were collected using the handheld Progeny equipment (Figure 1D) through their genuine bottles that included different sizes and shapes. The same settings given in the previous section were used. The actual ethanol and methanol concentrations for the methanol-spiked and the genuine samples used in this section of the study were determined using a gas chromatography–flame ionization detector (GC–FID). An Agilent 6890 series (Santa Clara, CA, USA) equipped with an HP G1513A autosampler and tray was used. The alcohol separation was carried out in an HP-FFAP (19091F-112, Agilent Technologies, Santa Clara, CA, USA) 25 m × 0.32 mm × 0.5 µm column with helium gas used as a carrier. The sample injection volume was 0.3 µL with a split ratio of 40:1 under a constant flow of 1 mL/min. The initial oven temperature was set at 50 °C for 4 min and then was increased at a rate of 2 °C/min to 60 °C and held for 2 min, followed by a temperature increase at a rate of 10 °C/min, reaching a final temperature of 120 °C, held for 1 min. The injector temperature was 150 °C, and the FID temperature was 250 °C.

### 2.4. Partial Least-Squares Regression (PLSR)

The spectral data were analyzed using multivariate data analysis software (Pirouette version 4.5, Infometrix Inc., Bothell, WA, USA). The data were mean-centered and then smoothed (S-G polynomial fitting algorithm with a 35-point window) prior to the multivariate analysis. Partial Least-Squares (PLS) regression is appropriate when the matrix of predictors has more variables than observations. Thus, PLS regression [16,17,18] was employed to develop reliable regression models to quantify ethanol and methanol levels using Raman and FT-IR spectra. For ethanol and methanol prediction, ranges of 850–910 cm^−1^ and 1020–1040 cm^−1^ were used, respectively. PLSR analyzes multivariate data with noisy and collinear variables by extracting a set of scores T by projecting the × block (spectra) on a subspace of latent variables that maximizes the covariance between the scores and the response (Y, i.e., methanol level), describing simultaneously the relevant × variance and correlating with the response(s) matrix [19]. This calibration process was done with a training set selected to contain the variability expected in future samples. The modeling error estimating future prediction performance of unknown samples was determined with the root-mean-square error of cross-validation (SECV) by using a full cross-validation (leave-one-out) procedure, where every sample is left out of the calibration once and subsequently predicted with the created calibration model. In order for the PLS calibrations to be accurate and reliable, the optimal number of latent variables leading to the minimum error during the validation stages (cross-validation) was selected to minimize the risk of omitting relevant variance (underfitting) or capturing not only the systematic information but also noise (overfitting), leading to poor predictions on new observations. Finally, an independent validation set including samples of known reference values that had never been involved in the training process was used to estimate the expected error for the prediction (RMSEP) of new and unknown samples. The validation step is essential to avoid both overfitting and underfitting [20]. Observations showed that unusual leverage and studentized residual patterns were identified as outliers and removed, as they can harm the quality of the predictions [21].

## 3. Results and Discussion

### 3.1. Quantification of Methanol and Ethanol Content with Reference Analysis

The alcohol analysis of Pisco spirits by GC–MS showed that the collection of samples from premium operations and small local distilleries yielded a wide range of ethanol concentrations, from 18 to 45%. Peruvian standards designate that Pisco must be distilled to have an alcohol content ranging from 38 to 48% alcohol by volume, meaning that producers cannot add water after distillation, which is standard for other spirits such as whiskey, rum, vodka, and gin [22]. The results indicated that 17 out of 171 samples fell outside the minimum alcoholic strength value (38% *v*/*v*) defined for standards of identity for Pisco [23] and should be labeled as a “diluted Pisco”. Puro Quebranta (single varietal, non-aromatic grape) spirits had 15% (10/67) of samples below 35% *v*/*v*, while the pure aromatics Pisco manufactured from Italia and Torontel grapes had 12% (5/43) and 8% (2/25) diluted samples that did not meet the standard of identity, respectively. Samples suspected of dilution were predominantly from small artisanal distilleries.

The methanol levels of Pisco spirits ranged from 10 to 130 mg/100 mL, reflecting the low methanol content of Pisco with respect to the legal limits of 0.4% (*v*/*v*) at 40% alcohol established by the European Union for naturally occurring methanol in fruit-derived spirits [24]. According to regulations, the methanol levels allowed in Pisco puro and green must range from a minimum of 4 mg/100 mL to a maximum of 100 mg/100 mL for non-aromatic grapes or 150 mg/100 mL for aromatic grapes [15]. Overall, 96% of the Pisco samples had methanol levels ranging from 10 to 55 mg/100 mL, with an average of 35 mg/100 mL, and none of the samples exceeded the methanol limit for standard of identity of Pisco.

### 3.2. Spectral Information

**Figure 2:**
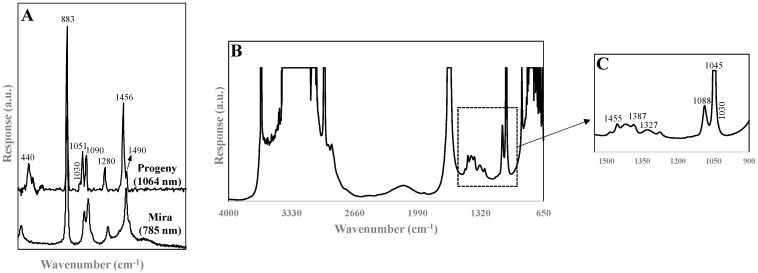
Typical Pisco spectra obtained from (**A**) handheld Progeny Rigaku (1064 nm) and Metrohm Mira M3 (785 nm) Raman instruments, (**B**) portable FT-IR device, and (**C**) fingerprint region of the Pisco spectrum given in (**B**).

The Raman spectra collected using the 1064 nm and 785 nm excitation laser had similar profiles, but the latter device required fluorescence subtraction functions, resulting in loss of spectral band definition (Figure 2A). Raman scattering of polarizable groups (symmetric vibrations of non-polar groups) showed six major bands present in all samples: the most intense band was centered at 883 cm^−1^ and was attributed to the symmetric C–C–O stretch of ethanol, a low-intensity band with a maximum at 440 cm^−1^ was assigned to the deformation vibrations of C–C–O and C–C, the bands at 1051 cm^−1^ and 1090 cm^−1^ were due to the stretching vibrations of C–C and the asymmetric vibration of C–C–O, the band at 1280 cm^−1^ was characteristic of the twisting vibration of –CH_2_– groups, and the band at 1455 cm^−1^ was associated with the asymmetric deformation of –CH_3_ [25,26]. A shoulder band at 1030 cm^−1^ was a key contributor for monitoring methanol levels and could be assigned to the C–O stretch [1,27]. Figure 2A,B show the complementary nature of characteristic fundamental vibrational modes of molecules collected using FT-IR and Raman spectroscopy. Because the FT-IR signal depends on a change in the dipole moment, hetero-nuclear functional group vibrations and polar bonds dominate the spectrum, especially the HOH bending vibration in water (2850 and 1650 cm^−1^) and the C–O and C–C stretching vibrations (1088 and 1045 cm^−1^). The last-mentioned bands were (1088 and 1045 cm^−1^) attributed to ethanol in the samples [28,29]. Similar to the Raman data, the shoulder band of the C–O stretching frequency at 1030 cm^−1^ was associated with the detection of methanol levels in Pisco [28]. The minor bands at 1460–1320 cm^−1^ were related to the O–H bending vibration of organic acids, mainly acetic acid, in Pisco [29].

### 3.3. Quantification of Methanol and Ethanol Content with Validated Regression Models

Multivariate analysis by partial least-squares regression (PLSR) was employed for developing predictive algorithms for the simultaneous estimation of ethanol and methanol levels in Pisco samples. Since the spectral patterns were dominated by the strong vibration modes of ethanol (Raman and FT-IR) and water (only for FT-IR), spectral transformation was applied to the Raman and FT-IR calibration models prior to the PLSR analysis to help resolve the subtle signal from methanol by lessening the spectral background noise. Accordingly, the best model performances were obtained by employing mean centering and smoothing (S-G polynomial fitting algorithm with a 35-point window) transformations for the Raman models (both for the Progeny Rigaku and Mira M3). In the FT-IR models, mean centering, divided by (sample-to-norm), and smoothing (35-point window) transformations were applied.

Table 1 shows the performance statistics of the PLSR models that were obtained from Raman (Progeny Rigaku (1064 nm laser) and Mira M3 (785 nm laser)) and FT-IR (Cary 630) instruments. The PLS models required few latent variables (2 to 3), determined by cross-validation (leave-one-out approach), to explain the relevant variance in the data matrix and minimize the risk of over-fitting (fitting random noise) or under-fitting (important unmodeled data) the model. The fitness or the strength of the model was evaluated with the coefficient of correlation (R) and the standard error of cross-validation (SECV). The best model performance for estimating the methanol content in Pisco samples was obtained using spectral data from the Progeny Rigaku Raman sensor, with cross-validated models giving 2.5 mg/100 mL and RCV of 0.94 (Table 1). These model performances were followed by those of the FT-IR and Mira M3 Raman instruments. The PLSR models for predicting methanol content using FT-IR spectral data provided SECV of 2.5 mg/mL and RCV of 0.90; on the other hand, the Mira M3 Raman provided SECV of 2.4 mg/mL and RCV of 0.89 (Table 1). The PLSR regression vector plots indicated that the band centered at 1030 cm^−1^, assigned to the C–O stretch of methanol vibrations, was responsible for predicting methanol content in the models. The cross-validated PLSR models to estimate the ethanol content provided similar performances in SECV and RCV with all three spectrometers. All sensors gave the same RCV of 0.97 and SECV of 2.5, 2.4, and 2.5 mg/mL by FT-IR, Mira M3 Raman (785 nm laser), and Progeny Rigaku Raman (1064 nm laser), respectively (Table 1). Raman PLSR regression vector plots indicated that the bands centered at 883 cm^−1^, related to the symmetric C–C–O stretch of ethanol, were accountable for the estimation of ethanol content.

**Figure 3:**
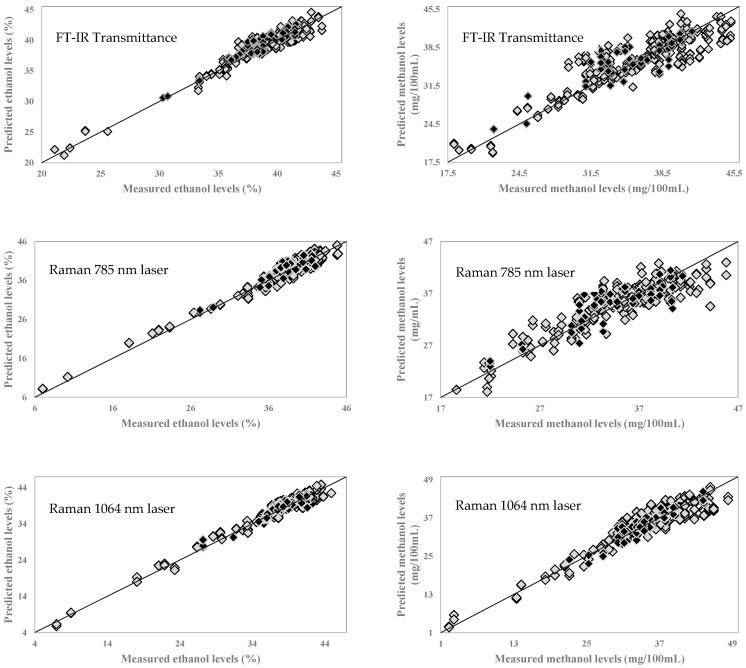
Partial least-square regression (PLSR) calibration and validation plots for ethanol and methanol levels in Peruvian Pisco samples using handheld Progeny Rigaku (1064 nm) and Metrohm Mira M3 (785 nm) Raman instruments and a portable FT-IR sensor. Grey diamonds represent samples in the calibration set, black diamonds represent samples in the external validation set.

The prediction accuracy of the generated PLSR models was evaluated using an external validation set. The external validation set consisted of randomly selected samples (20% of the total sample size), and the same samples were used as the validation set in all models (all the ethanol and methanol models with three different instruments) to provide a better comparison between different instruments. Table 1 shows the performance statistics of the generated external validation models; similar correlation coefficients (RCV and RPre) and error (SECV and SEP) were obtained. Figure 3 show the correlation plots that help visualize the relationship between the reference GC–MS values and spectroscopy-predicted ethanol and methanol levels in Pisco samples. Each correlation plot shows the dispersion of the external validation set samples within the calibration set range (Figure 3). The results in this study are superior or comparable with those of the alcohol and methanol content obtained in other studies that used benchtop or semi-benchtop Raman and FT-IR spectroscopy on various alcoholic beverages [29,30,31].

### 3.4. Quantification of Methanol and Ethanol Content through the Bottles

PLSR analysis was also used to determine the ethanol and methanol content from methanol-spiked samples. Spectra were collected only using the Progeny Rigaku instrument, since it gave the best methanol model performance in the previous section. Besides, because of the physical properties of the Mira instrument, a direct measurement (through-the-bottle analysis) could not be realized with the Mira instrument. A full cross-validated methanol and ethanol calibration models were generated for each alcohol for the measurements from glass vials and genuine Pisco bottles. The robustness of the models was evaluated using an external validation set that comprised 20% of the total sample size of each model. Table 2 shows the performance statistics of the PLSR calibration and external validation models. The full cross-validated training model of methanol from the glass vial was constructed by employing three factors and gave an RCV of 0.98 and a SECV of 110 mg/100 mL; on the other hand, the same component was estimated through the genuine Pisco bottles using four factors and gave an RCV of 0.98 and a SECV of 123.8 mg/100 mL. The methanol models’ robustness was confirmed with similar SEP and SECV and similar RPre and RCV values (Table 2). In recent studies, a handheld Raman spectroscope with a 1064 nm laser and a CCD array detector predicted the spiked methanol levels with root-mean-square error of prediction (RMSEP) from 140 to 340 mg/100 mL and Q2 from 0.86 to 0.98, through the genuine alcohol bottles, of alcoholic beverages, including Whiskey, Rum, Gin, and Vodka [1]. On the other hand, some researchers reported facing the problem of compromising the signal-to-noise ratio when analyzing alcohol through the bottle [32]; however, in the current study, that problem was not observed. Some possible explanations, including the Raman those researchers used, include the use of an 830 nm laser, since more extended wavelength lasers can introduce the fluorescence effect into the spectra; in this study, this possible problem was overcome by using a Raman with a 1064 nm laser. The other reason could be the different color of the glasses through which the tests were performed. The researchers reported that darker-colored glasses (green and brown) permit less light through the bottle; in the case of Pisco or some other type of alcoholic beverages (ouzo, raki, tequila, vodka), commercial samples come in clear bottles, which eliminates the light penetration problem.

The same spectral data were also used to predict the ethanol content in the Pisco samples from their genuine bottles; the model performance is given in Table 2. A full cross-validated training model from the genuine bottles employed six factors and provided RCV of 0.95 and SECV of 0.97%. The ethanol model’s robustness was indicated by their comparable RPre and SEP values (Table 2). Similarly, Kiefer and Cromfell (2017) analyzed single-malt Scotch whiskies (alcohol content 40–60%) through their bottles using a semi-benchtop Raman spectroscopy (785 nm laser) and predicted the alcohol content with a root-mean-square error of 0.44% [33].

## 4. Conclusions

Counterfeit alcohol is a global problem that has economic and socio-economic consequences. This study demonstrated the feasibility of a rapid prediction of ethanol and methanol levels in Peruvian Pisco using portable Progeny Rigaku Raman (1064 nm laser), handheld Mira M3 Raman (785 nm laser) sensors and a portable Cary 630 FT-MIR spectrometer. The data showed that the ethanol levels of 17 out of 171 samples were below the legal limit (38%), showing a possible dilution of the Pisco samples with water. Furthermore, the methanol content in the Pisco samples was successfully predicted by direct analysis through the glass containers after transferring to glass vials or through the genuine Pisco bottles (RPre from 0.97 to 0.99 and SEP from 98 to 103 mg/100 mL). The results of this study indicate that both Raman and FT-IR spectroscopies are promising tools to predict methanol and ethanol content in Pisco samples, while the Raman sensor with a 1064 nm laser (Rigaku Progeny) provided better performances overall and could be ideally suited for analyzing Pisco samples for its simplicity, reliability, and compatibility with traditional techniques.

## Figures and Tables

**Table 1:** Performance of calibration and validation models developed by using handheld and portable vibrational spectroscopy devices for the determination of ethanol and methanol levels in Peruvian Pisco samples.

Technique	Parameter	Calibration Model					External Validation Model			
		Range	N ^a^	Factor	SECV ^b^	R_cv_^c^	Range	n ^d^	SEP ^e^	R_Pre_^f^
FT-IR	Methanol	18.1–45.2	124	2	2.5	0.90	22.0–41.4	31	2.3	0.88
	Ethanol	21.1–43.8	124	3	1.0	0.97	30.3–41.6	31	1.0	0.96
Raman Mira M3 (785 nm)	Methanol	18.6–45.8	131	3	2.4	0.89	22.0–41.4	33	2.3	0.86
	Ethanol	7.0–44.9	136	2	1.4	0.97	27.2–42.0	34	1.4	0.94
Raman Progeny Rigaku (1064 nm)	Methanol	2.4–48.3	135	3	2.5	0.94	22.2–44.4	34	1.8	0.93
	Ethanol	7.0–44.9	132	2	1.3	0.97	27.2–41.6	33	1.2	0.95

^a^ Number of samples used in the calibration models, ^b^ standard error of cross-validation, ^c^ correlation coefficient of cross-validation, ^d^ number of samples used in external validation models, ^e^ standard error of prediction, ^f^ correlation coefficient of prediction for external validation.

**Table 2:** Performance of calibration and validation models developed by using a handheld Progeny Rigaku (1064 nm) Raman instrument for the determination of ethanol and methanol levels in spiked and pure Peruvian Pisco samples through their genuine bottles and glass vials.

Environment	Parameter	Calibration Model					External Validation Model			
		Range	N ^a^	Factor	SECV ^b^	R_cv_^c^	Range	n ^d^	SEP ^e^	R_Pre_^f^
Reading from Glass Vial	Methanol	10.3–2475.7	142	3	110	0.98	16.0–2543.9	36	103.0	0.99
Reading from Bottle	Methanol	10.3–2836.6	50	4	123.8	0.98	23.9–1441.9	13	97.7	0.97
	Ethanol	28.4–41.3	50	6	0.97	0.95	34.8–42.2	13	0.78	0.94

^a^ number of samples used in the calibration models, ^b^ standard error of cross-validation, ^c^ correlation coefficient of cross-validation, ^d^ number of samples used in external validation models, ^e^ standard error of prediction, ^f^ correlation coefficient of prediction for external validation.

## Data Availability

The datasets used for this study are partially available on request to the corresponding author.
